# Railroad Turnout Wear Diagnostics

**DOI:** 10.3390/s21206697

**Published:** 2021-10-09

**Authors:** Jerzy Kisilowski, Rafał Kowalik

**Affiliations:** 1 Faculty of Transport, Electrical Engineering and Computer Science, University of Technology and Humanities, 26-600 Radom, Poland; 2 Department of Avionics and Control Systems, Faculty of Aviation Division, Military University of Aviation, 08-521 Deblin, Poland; r.kowalik@law.mil.pl

**Keywords:** turnout, wear, railroad, diagnostics, railways

## Abstract

The article presents a few issues related to the technical condition of a railway turnout, an important element of the railway network where about 90% of railway accidents occur. In the first part of the article, the results of railway turnout wear are presented. A comparison of normal forces (in wheel–rail contact) in vehicle traffic on straight track without a turnout and normal forces occurring when a rail vehicle passes a turnout is presented. Then, turnout wear processes for selected speeds are presented. In the next part of the paper, the possibilities of using a vision system are presented, which, in combination with tools for image processing analysis, makes it possible to detect wear and distances between the key elements of a railway turnout. The main idea of the proposed online diagnostic system solution is to use the analysis of received images (photos) with the help of a vision system. The basic problem to be solved in the proposed system was to develop algorithms responsible for generating wear areas from high-resolution images. The algorithms created within the work were implemented and tested in the MATLAB software environment. The presented method is an original procedure for diagnosing turnout elements for each time instant. The proposed system is compatible with railway traffic control systems.

## 1. Introduction

The development of infrastructure investments on railroads contributes to an increase in the speed of traffic. Increase in speed of railroad traffic requires the managers of railroad infrastructure to increase spending on diagnostics and maintenance of railroad lines to ensure safety of railroad travellers. Railroad infrastructure is a complex system of dependencies, in which the weakest element decides the safety level of the whole system. As a result of analyses of threats resulting from the increase in speed of railroad traffic, the key element of the system is the technical and maintenance quality of railroad turnouts. Turnouts, which are crossings of tracks on railroad lines, influence safety and decide on the maximum speed of railroad traffic. The dynamics of rail vehicle passage through a turnout is discussed in publications [1,2,3,4,5,6,7,8,9,10,11,12,13,14].

Turnouts are an important element of railroad infrastructure, thanks to which it is possible to branch railroad lines or build stations. There are many types of designs, starting from simple turnouts, double (single-sided and double-sided), and ending with crossed turnouts. In most cases these are the constructions of which the geometry was fixed already several dozen years ago. The turnouts used in Poland are mostly characterized by the circular curve of the turning track axis. The description of these designs and the way of shaping them in the track system is described in publications [15,16,17,18,19,20,21].

Turnouts used on the network managed by PKP Polskie Linie Kolejowe S.A Warsaw, Poland have been classified according to types and basic geometric and construction parameters such as, among other things, radius of the turning track centreline, turnout bias, switch blade, cross member or turnout sleeper constructions. The planned construction of the high-speed railroad network and the issue of modernizing the national trunk lines make it necessary to look for a way of designing nonstandard geometrical solutions of railroad turnouts. One of the possibilities of solving this problem may be the construction of a numerical model of a turnout which, based on the given data, will automatically generate its design and provide the necessary information.

Correctly performed inventory and operational measurements of turnout geometry, junction, track brake, etc., followed by appropriate analysis and assessment allow to take appropriate remedial measures before the disturbing phenomenon or exceeding of the given operational parameter of the geometry of this device becomes dangerous for human life and health and causes traffic safety risk in rail transport or in the nearby environment [22,23,24]. Diagnostics of technical and operational devices includes turnouts, crossings, track crossings at turntables and alignment devices [25]. The construction of each railroad turnout is by definition a difficult construction, whose difficulty lies in the fact that the individual elements, with lengths of many meters, have to be assembled and then have to work in operation with an accuracy of 1 mm. The operated turnouts and track crossings are subject to cyclic measurements, which are a component of technical tests of turnouts. The scope and methods of measurements, permissible operation deviations, and documentation of diagnostic tests of turnouts are regulated by industry-specific instructions [26].

Turnouts are fixed on the elastic-plastic bed of variable stiffness and variable damping. They are followed by rolling stock traffic, rolling wheels, whose pressures depend on their geometry, travel speed, and axial load. Railroad turnouts and track crossings are loaded with variable values of vertical, lateral and longitudinal (horizontal) forces.

The current diagnostic procedures for switches are based on classical measuring equipment for the measurement of the track position in the plan and profile, e.g., track gauges, profile gauges, gap gauges, arrow gauges, measuring trolleys, and geodesic equipment. Classical diagnostics provides the possibility of detecting the resulting turnout damage and planning its repair. However, in the process of modernization and introduction of the newest standards in the maintenance of turnouts, it is useful to introduce testing procedures that make it possible to detect the defects at their early stage, ensuring the safety of the conducted traffic and maintaining the maximum speed of railroad traffic by preventing the defects that cause the necessity of introducing the reduction of the crossing speed. One of the methods for the early detection of turnouts defects is the use of image diagnostics for the three-dimensional monitoring of the examined elements, the other research method allowing for the early detection of defects is the analysis of dynamic effects caused by railroad traffic [27,28].

Turnout systems are one of the most important components of railway infrastructure and are equipped with a number of electromechanical devices. Therefore, the process of failure identification and diagnosis has attracted the attention of researchers and industry in recent years. There are three main approaches to identify system failures: feature-based, empirical and nonfailure models. In empirical feature-based approaches, special features are extracted to identify failures [29]. In model-based approaches, a failure is identified based on the amount of deviation of the collected signals from a predefined model [30,31]. Empirical feature-based approaches analyse the difference between the collected signal and a fault-free sample to identify failure [32,33]. Fault identification methods for turnout systems are summarised in [34].

The railway regulations currently in force [35], describe the rules and deadlines for visual inspection and technical testing of railway turnouts. However, they do not take into account many factors occurring in the maintenance process, which have a significant influence on the durability of railway turnouts. When carrying out the technical inspection of railway turnouts, the condition of all structural parts has to be checked and the detected damages and irregularities have to be written down in the logbook for the inspection of turnouts. Therefore, it is very often possible to find a general record of the type of damage found, especially when the observed changes are difficult to interpret or the description is too complicated. 

The solution to this problem can be online video diagnostics, which makes it possible to archive the defect image, analyse the damage course as a function of time or cumulative load and consult the occurring changes with an expert. A properly taken image of a defective component allows both a detailed description of the type of change and its comparison with the state found during the previous inspection. The diagnostic tests of turnouts also made use of an innovative measurement device in the form of an optical scanner for measuring the rail profile, used for the three-dimensional imaging diagnostics of cross bars, switch blades and retaining rods. Measurements using the scanner are conducted every 3 months on cross bars, switch blades and retaining rods. The tests are aimed at evaluating the wear and tear of switch components due to operation and early diagnosis of switch structure damages. The applied innovative rail measurement methods are compared with classical diagnostic methods to assess the research potential. The second stage of conducted research on turnouts on testing grounds is the research on dynamic interactions [36,37,38,39,40,41,42]. The tests are aimed at measuring the dynamic impacts generated by the railroad vehicle traffic in the turnout area and transmitted through the turnout to the track bed. The studies of turnout dynamics include the measurement of noise, vibration in the turnout, subgrade and the study of the deflection of the turnout caused by the train passage [43,44,45,46,47,48,49].

The results of tests of turnout geometry, profile from the first stage are compared with the results of dynamic tests from the second stage of the implemented research programme. The comparative analysis of the test results is aimed at the evaluation of the applied research methods for the early diagnostics of the turnout failures [50,51,52,53,54,55].

The efficiency and reliability of a railway turnout is influenced first of all by the component rails. Defects and damages of the rails (of different nature than in rail traffic), and above all, the rate of wear and tear increase are factors which directly translate into the durability of switch components and consequently into the life cycle cost (LCC). A characteristic phenomenon occurring in tram tracks is an intensive wear of rails in the switch point and the frog. This process, in a diverging line takes on a rapid and dynamic character. Cross-track components with considerable lateral wear pose a direct threat to the operational safety of rail vehicles. The interaction of wheels with rails, or rather the lack of their proper interaction, causes phenomena on their surfaces that are easy to observe. Wheelsets rolling on the rails are subjected to dynamic loads varying in time, which are the source of contact wear of wheels and rails. The abrasive wear causes deformation of external wheel contours, whereas the contact wear causes pitting and other types of damage on the wheel running surfaces and rail heads [56].

While aiming at the increase of safety with continuously decreasing maintenance staff and simplification of their work in railway stations, the notion of predictive diagnostics (dynamic diagnostics) and operational diagnostics should be more widely dealt with [57,58,59]. This paper presents the concept of computer diagnostics of a switch drive [60]. The point machine is an element that has a great influence on the safety of people and goods moving on the railway network. The switch drive plays an important role in railway station operation. Fast changing of switches is an important condition for efficient railway traffic control processes, therefore the switch drive is an important link in the railway traffic control system.

The dynamic response of a rail vehicle when passing through damaged turnouts and the detection of turnout damage has received much attention in the last decade. In [61], a high-speed turnout model was created and the dynamic response of the vehicle while passing through the turnout was analysed. However, turnout defect is not considered in this work. In [62], two methods for reproducing vehicle–turnout interaction are compared based on which the effect of vehicle impacts on a turnout in a city is studied. In [63] authors study the effect of turnout defects on wheel-rail interaction, the results show that turnout defects make vehicle vibrations more intense, but there is no further research on the change of vibration according to different defects. In [64], the causes of turnout defects are described and advice for their repair is listed.

From the presented analysis, we concluded that there is a lack of a system for conducting online turnout diagnostics in railway turnout diagnostic tests. This need results from very high wear of turnout elements when trains pass at high speeds (above 160 km/h). Such wear processes may cause the turnout elements to deform at any time. Such a phenomenon is eliminated by the proposed diagnostic system. 

Besides, the research on detection in turnouts mainly focuses on manual defect detection and detection by inspection car, how to detect turnout defects based on vehicle in service is not investigated in the available literature. As the manual detection method and track inspection method are expensive and inefficient, they are not sufficient for detecting turnout defects. All the reasons mentioned above motivate the work in this paper.

## 2. Materials and Methods

**Figure 1:**
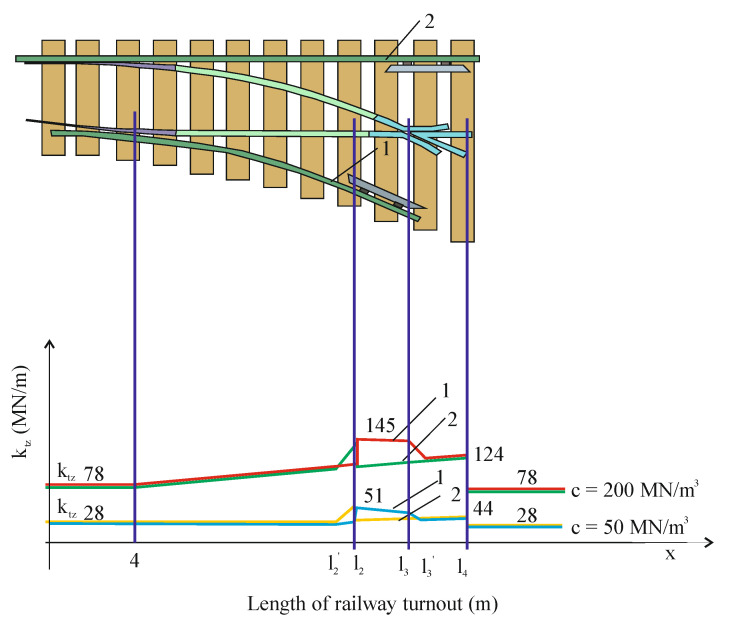
Diagram of variation of vertical stiffness coefficient of rail track geometry in the switch with different values of the substrate coefficient (real measurements on CMK Idzikowice): 1—internal track (with crossbuck), 2—external track [65].

Diagnostics of railroad turnouts, which are installed in track systems designed for high-speed train traffic, are an element that must be diagnosed in cycles included in national and European standards. The diagnostic element is important in turnouts because the turnouts are used by trains moving at speeds higher than 160 km/h. Turnouts for such speeds have a radius of 1200 m and more. The study was carried out for a turnout with a radius of 1200 m and with a fixed bow crossing. To understand the full answer to the task at hand, let us characterize the dynamic phenomena occurring in turnouts with high-speed train traffic. First, let us characterize the physical quantities occurring in the turnout and in relation to tracks without turnouts, curves, and intersections. Let us characterize the differences between a straight track (without turnouts, curves and crossings) and a turnout. The characterizing parameter is the susceptibility of the track. Tests have been carried out on specific objects [65], and their analysis was performed by determining the correlation coefficient between deformations and load. This coefficient varies from 0.91 to 0.98, which indicates that under these conditions the stiffness of the track can be assumed as constant and amounting to kt = 1.5 ∗ 108 N/m. The track stiffness at the turnout was also measured and varies along the turnout, as shown in Figure 1.

**Figure 2:**
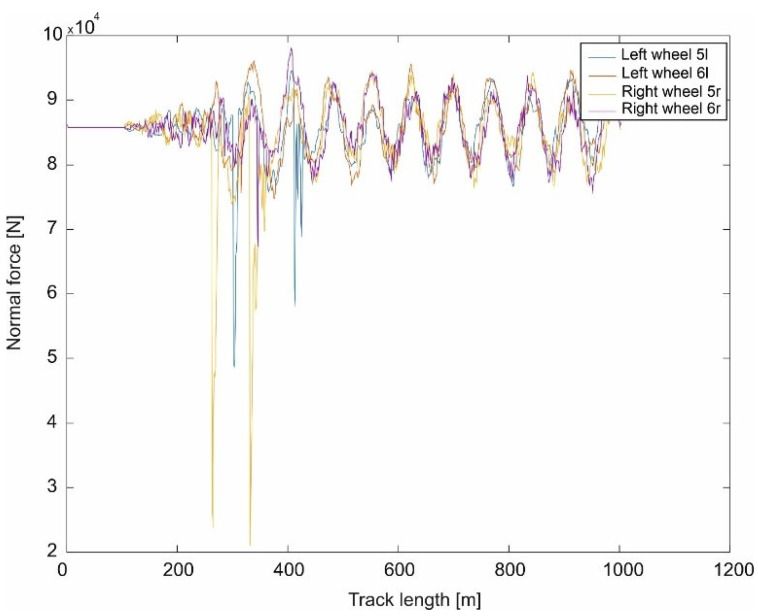
Normal force determined on a railroad turnout with constant stiffness.

Although the damping in the track is of a dry friction nature, it is assumed to be viscotic. For such a system (constant elasticity of the track and viscoelastic damping), a simulation was carried out for a mathematical model of a passenger car, and the variation of normal forces in the wheel–rail contact was obtained, varying around the value of static loads (weight per wheel in the vehicle) varying by the amount of relieving and adding loads (resulting from oscillations), as shown in Figure 2.

**Figure 3:**
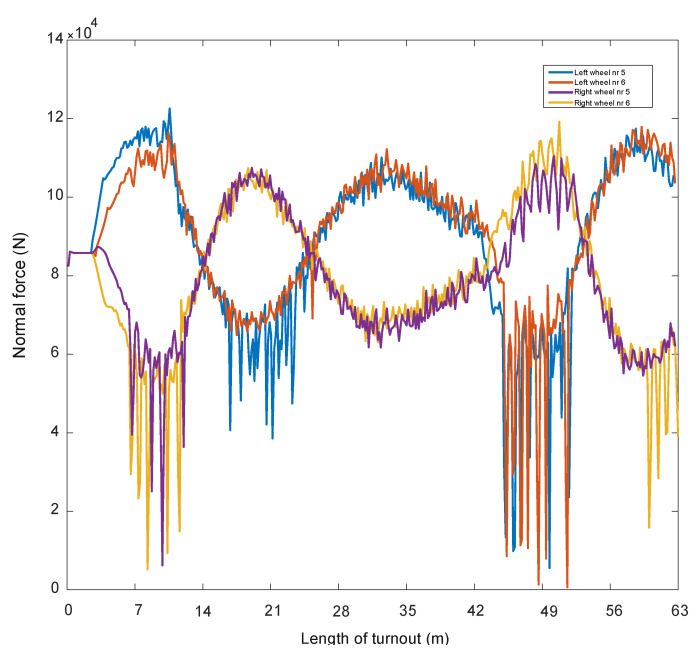
The course of normal force N at speed 350 km/h [68].

**Figure 4:**
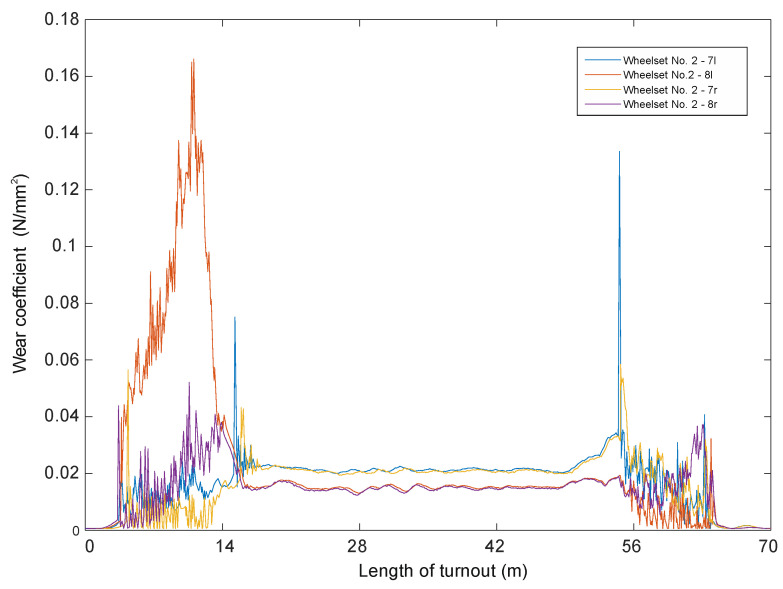
Wheel wear when passing through a turnout at 150 km/h [68].

**Figure 5:**
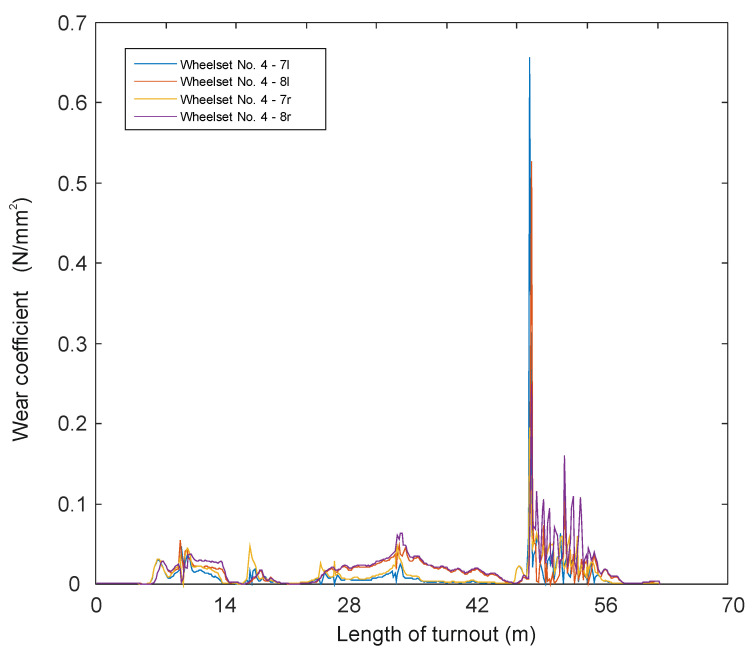
Wheel wear when passing through a turnout at 350 km/h [68].

In papers [66,67], a mathematical model of a passenger train moving on a track modelled as an Euler–Bernoulli beam was built. A simulation of the passage through the turnout was carried out using the computer systems Universal Mechanism, Matlab and IBBM Statistic. The passenger vehicle–track mathematical model for straight track traffic without a turnout includes a constant track stiffness (given above). However, when passing through a turnout, the track stiffness was assumed as shown in Figure 1. All parameters used in the passenger vehicle–track mathematical model at different stiffnesses were the same. A number of simulation results are presented and the magnitudes of the normal forces in the wheel–rail contact and wheel and rail wear are shown in Figure 3, Figure 4 and Figure 5.

When passing through a turnout, the normal forces in the wheel–rail contact show a large variation, especially in the region of the entrance to the spire and cross member. An example result for a speed of 350 km/h is shown in Figure 3. These quantities affect the size of the contact ellipses, and these quantities affect the wear process of the turnout elements and wheels. In this area of wear determination, [66,67] were used.

Wear quantities in the turnouts for 150 km/h and 350 km/h are shown in Figure 4 and Figure 5.

For speeds between 150 km/h and 250 km/h, there are wear increments in the initial parts of the turnout, but they are in the range of 0.006 mm, while in the area of the crossing for the speed of 300 km/h the maximum values are 0.25 mm and for the speed of 350 km/h-0.6 mm. It can be seen from the presented results that the passage of a rail vehicle with the speed of 300 km/h and 350 km/h causes large wear of wheels in the area of cross member (cross member with fixed bow). 

**Figure 6:**
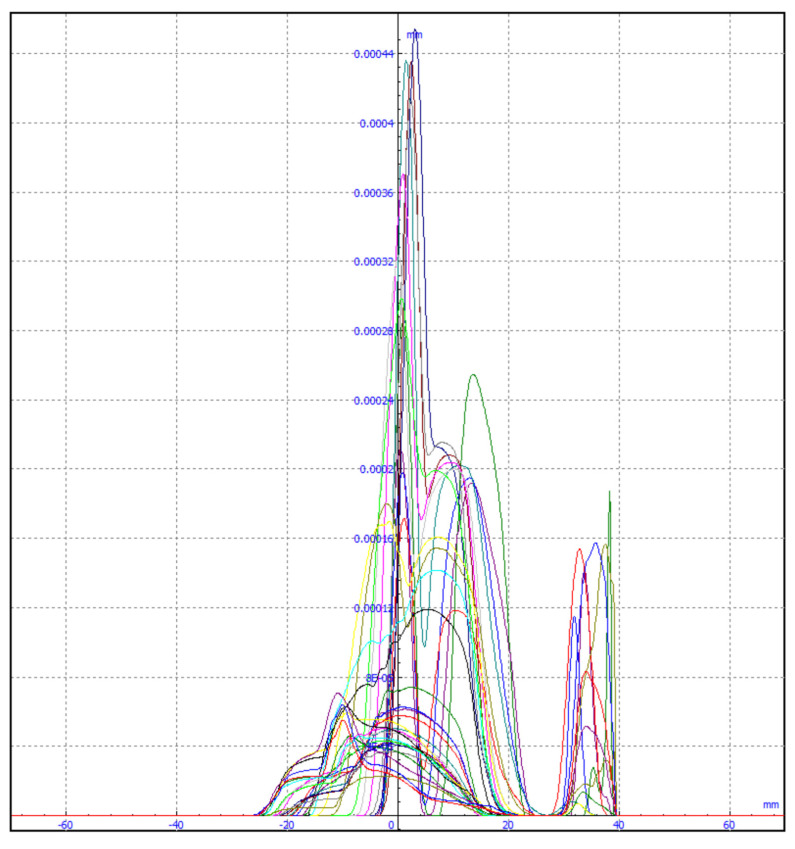
Wear of the rail by the left wheel of the first set when the rail vehicle passes through the turnout at 200 km/h on straight track [68].

In [69], a simulation of a passenger train passing through a turnout 20,000 times was carried out, recording the wear of the turnout elements of the railroad track where the crossbuck was located. Figure 6 shows the rail wear for 20,000 train passes through the turnout at 200 km/h. These facts indicate that the wear of the turnout elements depends on the speed which makes it necessary to study the condition of these elements online (due to large wear processes).

The results presented are for wheel and turnout wear as the sum of wear of all elements in the turnout. The proposed system will take into account wear measurements of characteristic turnout elements. 

The development of infrastructure investments on railroads contributes to an increase in the speed of traffic. Increase in speed of railroad traffic requires from manager of railroad infrastructure to increase spending on diagnostics and maintenance of railroad lines to ensure safety of railroad travellers. 

Turnouts are an important element of railroad infrastructure, thanks to which it is possible to branch railroad lines or build stations. There are many types of designs, starting from simple turnouts, double (single-sided and double-sided), and ending with crossed turnouts. In most cases these are the constructions the geometry of which has been fixed already several dozen years ago. The turnouts used in Poland are mostly characterized by the circular curve of the turning track axis. The description of these designs and the way of shaping them in the track system is described in [70].

## 3. Measurements and Key Parameters in Turnout Technical Condition Assessment

All turnouts shall be subject to technical inspection, which shall include evaluation of the technical condition of all structural parts and the geometric system, the efficiency of their operation, the condition of maintenance, and measurement of the track gauge, track gauge and grooves at the locations indicated on the technical inspection sheets.

Turnout diagnostics include [70]:

- Visual inspection conducted visually in order to determine if there are any cracked, chipped or damaged parts in the turnout as well as other defects or deformations that may affect the proper operation of the turnout;
- Technical tests (inspections), which include:
  − Visual inspection of the turnout;
  − Assessment of technical condition of all structural parts and geometric layout;
  − Assessment of correctness of operation of moving parts.

**Figure 7:**
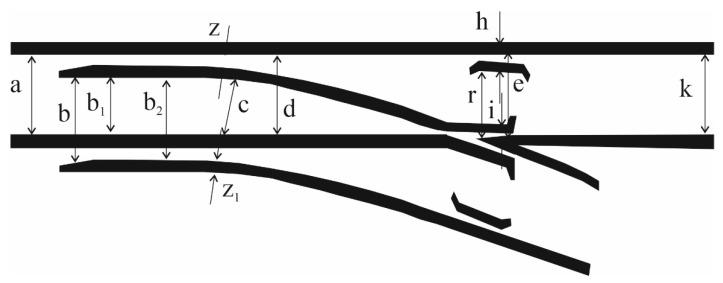
Measurement scheme of width and grooves of the turnout [1]: in the pre-spire contact a, in the firing pin blade b, in the firing pin seat c, in the middle of the turnout d, before the throat s, in the cross member e, and the distance of the leading edge of the steering wheel from the closer edge of the bow f and the width of the groove in the firing pin socket g, at the steering wheel h, in the cross member i, in the throat g, in the firing pin housing z. [70].

The key determinants considered in the process of evaluating the wear and condition of turnouts are related to the discrete measurement of the track gauge and grooves at a specific location of the railroad turnout (Figure 7), and monitoring the qualitative condition of the needle and cross member. 

Figure 7 shows the deviation measurement points for a railway turnout and their acceptable values are shown in Table 1 and Table 2.

Table 3 presents the key values that should be monitored to ensure that the technical state of a railroad turnout is within the standards defined in the instruction for the inspection, technical testing and maintenance of turnouts elaborated by PKP Polish Railway Lines, Warsaw, Poland.

In the process of operation of the diagnostic system it is necessary to properly define the tested object. In the analysed case it is the main (main line) track and the diverging line. The final results should be saved as a graphic file. During the operation of the diagnostic system for a railway turnout, all the technical data collected will be forwarded to the control centre responsible for monitoring the train control systems. In the track gauge measurement locations, the difference in track gauge height is also measured. In turnouts designed for trains moving at speeds exceeding 150 km/h, additional measurements of the groove and track gauge are performed. The system monitors in real time the curvature of turnouts with a radius exceeding 1000 metres. The results of the measurements carried out are compared with the nominal values and we check whether the permissible values of deviations and wear of the elements constituting the switch are not exceeded. The currently used permissible wear limits do not in any way take into account the differences in turnout operation, especially in train speeds and accumulated loads. Turnouts operated on side tracks should meet the same requirements as turnouts installed on main tracks. The principles of the online railroad turnout diagnostic system are presented in [71]. It should be pointed out that the process of the real analysis of the turnout image makes it possible to record a lot of information, which can be useful for the damage analysis during the next technical tests. Correctly performed and described structure of the obtained results of the damaged turnout element may in the future be a perfect material useful in the assessment of the phenomenon. In practice, online image diagnostics should be used from the moment of laying the new turnout, even if no defects have been detected. It is then necessary to take some general pictures, e.g., of the switch and resistor, crossbuck, crossbuck bow, anticrawl device, and the view of the turnout in relation to the adjacent tracks and other turnouts. Then they can be uploaded to the system database and used for comparison processes. An important problem in the diagnostic process is the classification of the detected defects. 

## 4. Railway Turnout Diagnostic System Architecture

The current turnouts diagnostics procedures are based on the classical measurement apparatus for the measurement of the track position in plan and profile, e.g., track gauges, profile gauges, gap gauges, arrow gauges, measuring trolleys, and geodetic devices. Classical diagnostics provides the possibility of detecting the resulting turnout damage and planning its repair. However, in the process of modernisation and introduction of the latest standards in the maintenance of turnouts, it is advisable to introduce testing procedures that make it possible to detect faults at their early stage, ensuring the safety of the traffic being carried out and maintaining the maximum speed of railway traffic by preventing the faults that make it necessary to introduce the reduction of the crossing speed.

One of the methods for early detection of turnout failures is the diagnostic system proposed by the authors of this paper for three-dimensional diagnostics of the tested elements, and the second research method allowing early detection of failures is the analysis of dynamic effects caused by railway traffic.

**Figure 8:**
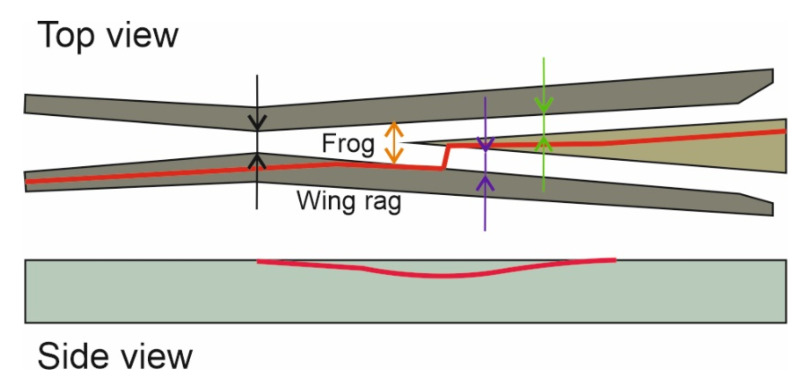
Measurement system of the railroad turnout cross member characteristic quantities.

The tests of turnouts are divided into two parts. The first part of the research programme comprises the tests of turnout geometry in plan and profile carried out using classical measurement techniques in accordance with the accepted standards and with the use of the innovative diagnostic system. The diagnostic tests of switches also make use of an innovative measurement device in the form of an optical scanner for the measurement of the rail profile, used for the three-dimensional image diagnostics of crossings, switch blades and retaining rods. Measurements using the scanner are conducted every 3 months on cross bars, switch blades and retaining rods. The tests are aimed at evaluating the wear and tear of switch components as a result of operating conditions, as well as at early diagnosis of switch structure failures. The applied innovative rail measurement methods are compared with classical diagnostic methods to assess the research potential. The second stage of conducted research on turnouts on testing grounds has the character of dynamic impact tests (Figure 8). The aim of the research is to measure the dynamic effects generated by railway traffic in the turnout area and transferred by the turnout to the track bed. The investigations of turnout dynamics include the measurement of noise, vibrations in the turnout and subtrack as well as the examination of turnout deflections caused by the train passage. 

The assessment of geometrical accuracy of a turnout, which is a system of two rail tracks, a switch blade and a cross member arranged in a fixed distance between them, is carried out on the basis of diagnostic tests of the following basic values (parameters) specified in Figure 7:

(a) Track gauge (Figure 8a,d);
(b) Distance of the stub axle to the spire;
(c) Distance of the stub rail to the bow of the cross member;
(d) Distance of the wing rail to bow of the cross member.

Diagnostic systems are used to identify the wear of rail components of a railway turnout and the strength qualities of the rails require the loading of reference photos from the structural components of the railway turnout. The photos from the initial state are used for comparison with the photos obtained in individual stages of railway turnout operation. In the diagnostic process, time courses of selected values-measured on-line-are used to extract significant events from the point of view of railway turnout wear diagnostics. These values are analysed. The most frequent methods of analysis include various types of transformations, which allow for appropriate interpretation of the signals and extraction of the most important information concerning the course of the process.

As a result of the operations, specialised software determines the deviations arising on the analysed elements. The system is enriched with optoelectronic distance sensors which will be responsible for measuring such distances as:

− The track width over the entire turnout length;
− Distance of the leading edge of the steering wheel from the closer edge of the bow
− Width of the steering wheel groove;
− Width of the groove at the crossing;
− Difference of track gauge height at the gauge measuring point.

**Figure 9:**
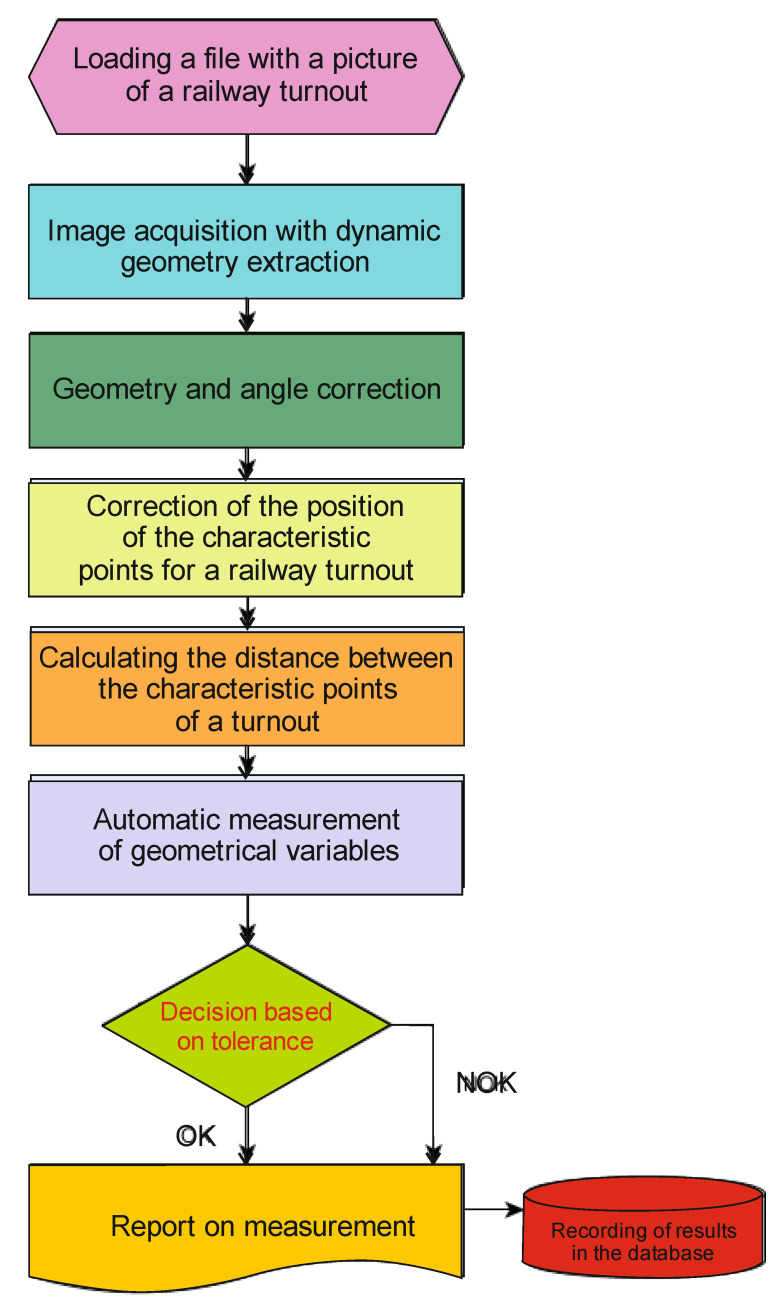
Procedure for determining geometrical parameters of a railway turnout in an online diagnostic system.

The innovative diagnostic system for railway turnouts, the architecture of which is shown in Figure 9, will be used for measurement of the track gauge and height difference of rail tracks (difference in cant) [71].

Measurements of the mentioned quantities characterizing the geometrical state of the turnout, whose detailed rules and organization are specified in the instruction [28], are performed as direct measurements using portable measuring devices (the so-called manual measurements and self-registering electronic track gauges) or as indirect measurements using measuring vehicles. Direct measurements are carried out in a point method (discontinuous), i.e., at fixed distances (with measurement steps), e.g., every 5 or 10 m with a so-called hand-held tachometer or in a method referred to as direct continuous measurement, measuring with a self-registering electronic tachometer fixed parameters registered automatically at distances of a few mm. Indirect measurements are more effective compared to direct measurements because they make it possible to make a complex assessment of the geometrical condition of the track on long stretches of the line by carrying out measurements with special trolleys and measuring wagons at speeds up to 250 km/h in the conditions of the track loaded with a vehicle, i.e., in conditions close to the real operating loads. The entire predefined measurement process was included in the developed online diagnostic system.

On the basis of the recorded measurement results, the assessment of the track geometrical condition and wear is performed by determining the deviations and comparing them with the permissible values.

The evaluation of the geometrical condition of a railway turnout and its wear can be carried out either for selected parameters (e.g., for short sections (switch blade or crossing blade area) due to particularly bad condition of such parameters), or for longer sections in a comprehensive manner (the whole railway turnout), taking into account all of the above-mentioned four parameters characterising the geometrical condition. In case of a comprehensive assessment of wear, synthetic (cumulative) indicators providing a more objective assessment shall be used compared to the assessment of single parameters.

The manual measurement of the geometrical values characterising a railway turnout with the use of, for example, a camera image is a fast and accurate process. The measurement result may be distorted by the subjective assessment of the person making the measurement. The attention should be paid to external factors such as a strong light source, changing temperature or low-frequency vibrations that can significantly falsify the obtained results. Proper positioning of the measured object is also of great importance. The cameras, when properly calibrated, can be used for photometric and geometric measurements. All the problems mentioned above can be minimised by using an automated online turnout visual diagnostic system.

The operator of the diagnostic system chooses the area (the turnout region) where the analysed component of the turnout to be measured is located, then he chooses one of several dozen measurement tools (e.g., the measurement of the distance between the switch point and the frog, the distance between any points of the wing rails and the crossing bow, the wear of switch components), defines the limits within which the result must be found and gives a name to the measurement result, which unambiguously defines the measurement region of the turnout geometrical value. Mathematical operations are also available to facilitate nontypical or difficult measurements. All settings, such as camera position, exposure times for individual images, areas of interest, selected postmeasurement tool, measurement tolerances, calibration, are saved in a graphic file with the extension png as metadata. Each of the created final images can be freely edited or deleted. The algorithm for determining geometric values of railway turnout is presented in Figure 9. 

Online diagnostic tests of a railway turnout can be divided into two basic groups according to their subject: geometrical tests of tracks included in a railway turnout and tests of railway turnout wear.

Railway turnout diagnostic tests, irrespective of their subject, are carried out according to the same general rule, consisting in the following sequence of actions taken:

- Selection of the test object, i.e., the quantity belonging to one of the two mentioned groups;
- Selection of the method and measuring equipment appropriate to the selected quantity (parameter) and to the quantitative range of the planned measurements;
- Execution of measurements and determination of the set of their results;
- Processing of the measurement results in order to determine the set of deviations, i.e., the difference between the measured and nominal values of the determined parameters;
- Qualification of deviations to the set of unacceptable deviations (defined for particular parameters in the regulations) and acceptable with their possible division into established classes;
- Determination of assessment of track condition and forecast of development of permissible deviations;
- Qualification of the examined track section wear to the specified scope and deadline for repair activities—i.e., planning the repair.

The process of track structure maintenance and its part, which is a diagnostic process, are carried out on the PKP network on the basis of regulations, and in particular on the basis of instructions of PKP PLK S.A. [28].

Geometric investigations with online diagnostic system of a railway turnout are aimed at determining the geometric distances in the area of the switch blade and crossover, which significantly influence the assessment of a technical condition of a railway turnout and thus the conditions of its operation. The driving calmness, traffic safety and durability of turnout components depend on geometric distance between subassemblies of a turnout.

**Figure 10:**
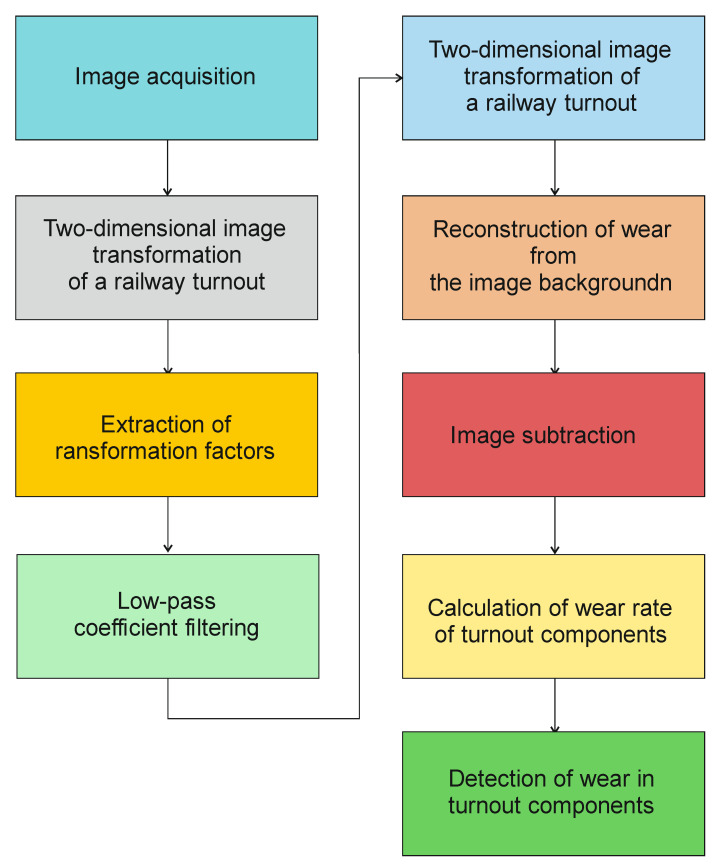
Procedure for determining the wear of a railway turnout in an online diagnostic system.

In the system developed by the authors, vision diagnostics was used together with an image processing algorithm in order to determine the most worn out elements. The algorithm determining the wear of a railway turnout is presented in Figure 10.

A photograph depicting a railway turnout can be treated as a process of segmentation (division) of the image into its certain fragments (spire, cross brace and rails). There are many segmentation methods that can be divided into four groups: those that detect edges in the image, on the basis of which segmentation is performed, whereby gradient methods are most often used to detect edges; those based on first-order statistics, whereby, in order to extract elements in the image, the brightness distribution for a single point is examined using mean value, variance or entropy; those based on second-order statistics (the so-called Haralick measures), whereby, in order to extract elements from the image, the relationship between the brightness intensities of two points at a fixed distance from each other is examined; multiresolution analysis, using Gauss-Laplace pyramid or wavelet transforms. 

**Figure 11:**
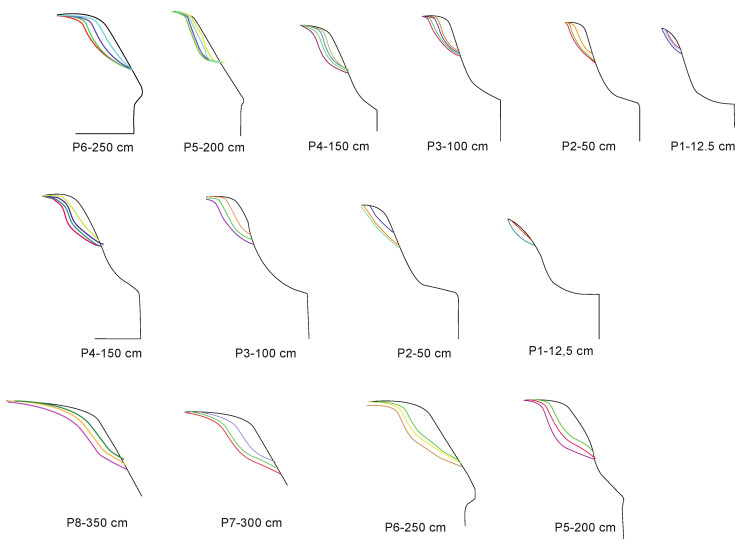
Spire wear for a railroad turnout with a radius of 1200 m at different operating periods.

**Figure 12:**
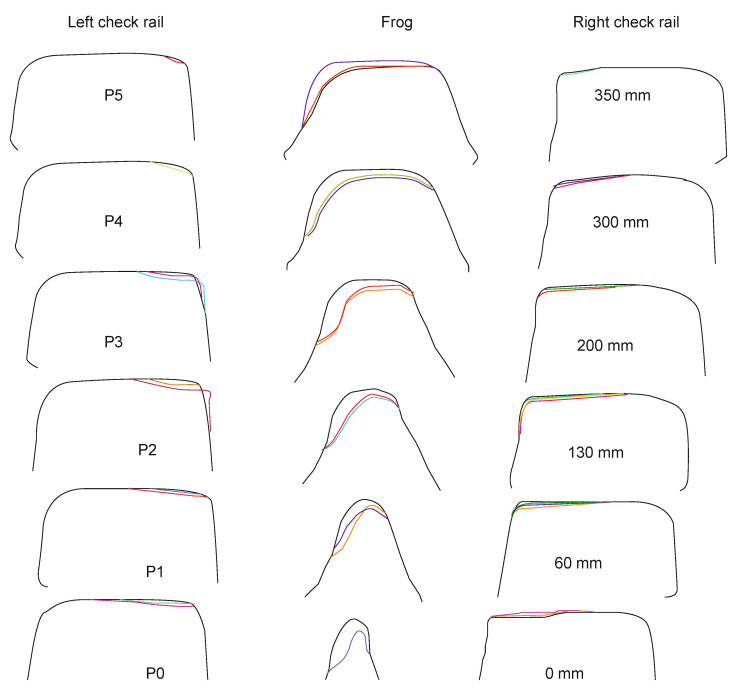
Cross member wear for a railroad turnout with a radius of 1200 m at different operating periods.

The authors tested each of these groups for their suitability for railway turnout operation and wear. Due to the fact that the head of the rail, the switches point and the frog of the crossing can contain both impurities and surface defects of different sizes, the application of methods belonging to the first group does not give satisfactory results. The application of the method using an entropy filter (belonging to the second group) for crossbuck extraction yielded satisfactory results, nevertheless, due to its time-consuming nature for high-resolution images (in the case analysed, 4096 × 3112), it was decided to find another solution allowing significant acceleration of the algorithm of railway turnout geometrical lines and wear. The results from rail and needle wear are shown in Figure 11 and Figure 12.

Diagnostic tests were performed in ordinary turnouts with a radius of 1200 m. 

The best methods from the point of view of both accuracy and speed of segmentation turned out to be those based on the two-dimensional discrete wavelet transform (2D DWT), belonging to the fourth group. With this transformation, it is possible to analyse an image at so-called multiple scales, whereby a smaller scale image contains elements of larger size than a larger scale image. This property can be used to reduce the influence of dirt and surface defects in the rail fragment on the accuracy of the extraction process.

### Mathematical Model for Distance Determination Using Camera and Optoelectric Sensor

The created online diagnostic system is based on the fact that a defined area of the railway turnout is registered with a high resolution camera during the survey. The acquired image is analysed for the existence of predefined characteristic elements in it, which are the components of the railway turnout and the specific components of the crossing and the switch blade. These components are searched for in the image to determine distances at characteristic points and to determine operational wear. As the railway vehicles repeatedly pass over the turnout, the distance between the wing rails and the frog bow changes. Identical changes occur between the needle and the switch blade. Based on the image made at the initial stage of railway turnout operation and comparing it with the current image, it is possible to determine the distances between individual components of the railway turnout and the wear of the rails. The diagnostic system consists of: a camera, laser position markers, PC computer equipped with an image acquisition card, digital input/output card and a dedicated computer application. 

The use of an automatic, stationary online diagnostic station based on the vision method eliminates all the disadvantages of manual measurement, and it meets additional requirements such as:

(1) No need to install any additional structural elements on the locomotive and to use specialised diagnostic equipment—so-called noninvasive measurement;
(2) The possibility of remote operation of the measurement process from a room away from the railway turnout—so-called measurement with online visualization;
(3) Automation of the measurement process—the measurement process can be performed by one person thanks to the use of signals available through wireless infrastructure. The results are represented in a form accessible to the operator and unambiguously classified as compliant with the standard or not.

The use of a mast is proposed, which allows the camera to be fixed and also allows the laser sensor to be always in the same position under the camera, which ensures the right angle of image acquisition, i.e., the accuracy of the reading.

**Figure 13:**
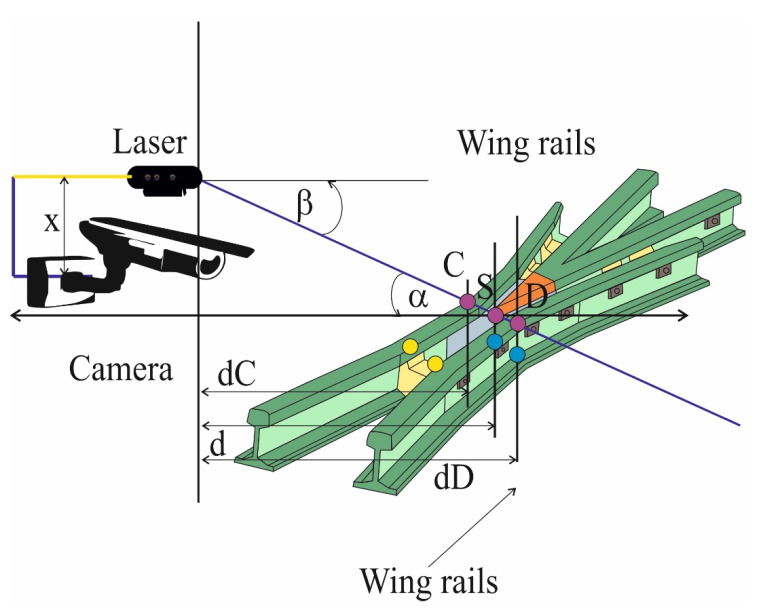
Method for determining the gap width between the resistor and the firing pin.

Apart from the vision apparatus in the form of a camera, an important element in the process of determining distances characteristic for the bow of a switch blade and its other components is an optoelectronic sensor. In the situation of defining the mutual relations in the form of mathematical equations, it is required to determine the position of the laser in the image of the vision apparatus and, on the examined elements of the turnout, determine the distances between the camera and the analysed object, which can be either a spire or a cross beam [71]. The general model of the system for which the mutual mathematical relations were determined is shown in Figure 13.

According to the adopted designations, *dC* denotes the minimum value of the distance to the monitored set of elements included in the structure of the railroad turnout. The defined parameter can be calculated from the equation:

(1) $$dC=\frac{x}{\tan\left(\alpha \right)+\tan{\left(\beta \right)}^{\prime }}$$

Then, after determining *dC*, the parameter *dD* was determined, which was then used to estimate *dD* by determining the length to the maximum point monitored by the diagnostic system:

(2) $$dD=\frac{x}{\tan\left(\beta \right)+\tan{\left(\alpha \right)}^{\prime }}$$

In the next step, the distance between the monitored object and the video camera was determined, assuming *dC* < *d*< *dD*. The mentioned parameter can be determined from the relation:

(3) $$dD=\frac{x}{\left(\tan\left(\alpha \right)+\tan\left(\beta \right)\right)-{\left(2p\cdot \tan\left(\alpha \right)\right)}^{\prime }}$$

The variable *p* is an auxiliary quantity that represents the proportional value between the distance measured by the laser of the tested turnout component and the distance in the horizontal direction, which can be calculated from the expression:

(4) $$p=\frac{l}{l^{\prime }}$$

The parameter l appearing in the formula (4) is responsible for the real value of the distance between the camera and the element of the railway turnout taking into account the size *d*, while *l*′ is the real length between the point indicated on the monitored object by the laser and the edge of the image on the length d. The value of *l* can be determined from the mathematical relation:

(5) l=2d⋅tan(α),

The presented mathematical model is correct under the following conditions:

(6) β>α and 0<p≤1,

## 5. Turnout Wear under Operating Conditions

**Figure 14:**
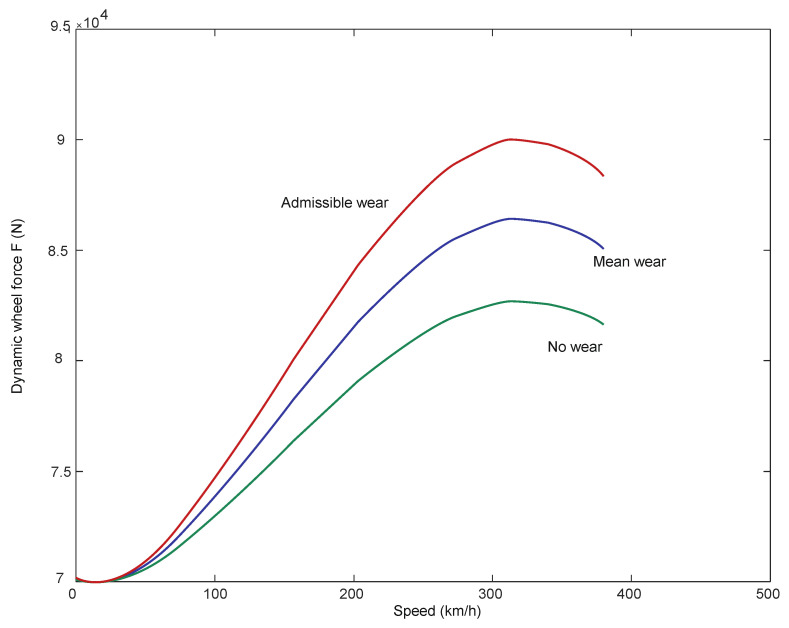
Wear on railroad turnout rails from rail vehicle speed.

The durability of railroad turnouts depends on the service properties of the material, turnout design and operating conditions. A comprehensive analysis of the issues related to the broadly understood durability requires the determination of the wear intensity of the turnouts in the operating conditions. Figure 14 shows the dependence of the wear function on the rail vehicle speed.

Wear measurements of the cross members consisted in comparing the measured profile with the master profile of the UCI60 block section. The solid line denotes the master profile, the fine dotted line the profile obtained during the first test, while the dashed line represents the profile measured during the last test. Only the results of the first and last measurement cycle are included in this paper due to the relatively small increase in wear between successive measurements. The tests were performed in three measurement planes, approximately 10 cm, 60 cm and 110 cm from the beginning of the cross beam. The gauge was based in the turnout during the measurement cycle, using a special beam as a base. After setting the gauge in the required position, the operator led the spherical measuring tip along the controlled surface, and the trajectory was recorded in the electronic memory of the device. The measurements of wear of pad sections in operating conditions, installed in turnout cross bars, showed uneven wear of the rolling surface profile along the length of the cross bar bow and its plastic flattening. The greatest wear occurs at the bow of the cross beam at the point where the greatest load occurs, and it decreases as the distance from the bow increases. Wear of such character is caused by momentary increase of dynamic load acting on the small rolling surface of the bow. This causes its intensive wear and plastic flattening due to impacts from the wheels of the rail vehicle.

### 5.1. Wear Functions

The purpose of the diagnostics of the superstructure elements is to determine their technical condition of a railroad turnout, wear and tear, and possible scope of works necessary to maintain the turnout in a given class. The assessment of turnout elements is carried out during the visual inspection and technical testing (inspections). In order to improve the process of turnout diagnostics, the authors of this paper have proposed the concept of an online diagnostic system that will analyse the wear of all switch components in real time. Currently, the results of inspections and tests of the track superstructure elements must be recorded in the documentation of the technical condition of the track superstructure. In the online diagnostic system the data will be analysed in defined periods of technical tests and compared with the image acquired during the previous diagnostic tests. Then the obtained current image will be compared with the pattern and on this basis the current wear of switch elements will be determined.

Diagnostics of a railroad turnout includes assessment of the condition of rails, rail resistor, switch blade and switch bow.

Diagnostics of rail and other components of a railroad turnout include:

- Visual detection and measurement of external defects and damages;
- Measurement of vertical, lateral and angle wear of the rail head, resistor, firing pin and cross beam;
- Measurements of wavy wear on the running surface of the rail.

The rail wear limit parameters are shown in Table 4.

### 5.2. Turnout Diagnostics System in Online Mode

Laser triangulation is used in imaging the shape and dimensions of an object. The feature points of the spire and crossbuck are illuminated by a laser line generator from one direction, while the camera captures an image of the indicated objects on the railroad turnout from another direction. The laser line that appears on the surface of the railroad turnout elements is recorded by an image sensor equipped with the camera. On this basis, and using parameterized algorithms implemented in the camera’s microprocessor, the height and distance of each characteristic point defined on the spire and crossbar in cross-sectional view is determined by analysing the course of the laser line in the sensor image.

The result of the measurement is a wear profile that contains one value for each of the measured points along the feature points of the turnout, for example, the width between the bow of the cross member and the wing rail. To measure the third dimension of wear on a railroad turnout, we need to add an additional laser sensor on the opposite side from the camera and laser sensor set. Therefore, the result of such a scan of a railroad turnout is a set of profiles, where each profile contains a cross-sectional measurement at a specific point along the direction of rail vehicle travel.

The wear profile values generated by the 3D camera are not calibrated, that is:

- Values of the distance between the bow and the wing rail (z coordinate) are given as a number, depending on the number of characteristic lines defined on the switch blade or the crossbar or pixels located on the camera sensor;
- Position of the point along the cross section (x coordinate) is given as a number indicating the sensor column in which the turnout characteristic point has been measured;
- The position of the point along the direction of movement (y coordinate) is represented by, for example, the consecutive measurement number and is recalculated from the known speed of the rail vehicle or measured directly.

In order to obtain calibrated measurements, i.e., coordinates and heights in millimetres, the camera image sensor coordinates must be converted to a real *x, y, z* coordinate system. This conversion depends on many factors, e.g., the distance between the camera and the railroad turnout, the angle between the camera and the laser beam, as well as the characteristics of the lens. This transformation is described by a sequence of simple formulas with a very limited number of open parameters, and parameterized in two steps. First, the lens correction is performed, followed by the perspective correction. Perspective mapping is also called homography. The lens distortion is corrected based on a standard polynomial radial model to transform the sensor coordinates (u, v) to the lens plane (u′, v′):

(7) $$\begin{array}{l} u^{\prime }=u+u_{0}\left(c_{1}r^{2}+c^{2}r^{4}\right)+2c_{3}u_{0}v_{0}+c_{4}\left(r_{2}+2u_{0}\right)\\ u^{\prime }=u+u_{0}\left(c_{1}r^{2}+c^{2}r^{4}\right)+2c_{3}u_{0}v_{0}+c_{4}\left(r_{2}+2u_{0}\right)\\ u_{0}=u-u_{c}\\ v_{0}=v-v_{c}\\ r=\sqrt{u_{0}^{2}+v_{0}^{2}} \end{array},$$

where (u_0_, v_0_) is the optical centre of the sensor and c_1_, c_2_, c_3_, c_4_ are parameters that determine the lens distortion. 

This model is sufficient for most standard lenses, except for wide-angle fisheye lenses. The optical centre of the turnout matrix is one of the so-called intrinsic parameters, which are those that depend on the design features of the intrinsic parameters, which are those that depend on the design features of the camera. Other typical internal parameters that can be included in the model are imperfections in the assembly or manufacturing of the transducer. Evaluation of distortion coefficients is done experimentally, based on scanning a flat disc element. Since the flat target does not contain information along the flat surface, it is not possible to measure distortion along the test object. Therefore, it is important to expose the object at different inclination.

Perspective correction, which is the mapping of the lens plane to the plane of the object under study (a turnout photo obtained at the initial stage of use), on to which the results obtained from the diagnostic system are superimposed, is defined by uniform coordinates:

(8) $$\left[\begin{array}{c} X\\ Z\\ s \end{array}\right]=H\left[\begin{array}{c} u^{\prime }\\ v^{\prime }\\ 1 \end{array}\right]=\left[\begin{array}{ccc} b_{11} & b_{12} & b_{13}\\ b_{21} & b_{22} & b_{23}\\ b_{31} & b_{32} & b_{33} \end{array}\right]\left[\begin{array}{c} u^{\prime }\\ v^{\prime }\\ 1 \end{array}\right],$$

The real coordinates (x, z) are obtained by introducing a standardization factor s:

(9) $$x=\frac{X}{s},y=\frac{Y}{s}$$

Theoretically, all nine coefficients of the H matrix can be determined by a measurement based on a single scan of a sawtooth target (railroad turnout components) whose dimensions are known. In practice, however, multiple scans are performed to cover the entire field of view of the camera. 

## 6. Wear Detection Algorithm in a Railroad Turnout Diagnostic System–Online

Each railroad turnout in the process of designing and manufacturing parts and then during their assembly acquires properties that can be described by a certain set of physical characteristics. These features are sometimes called turnout characteristic parameters or condition parameters. The values of these features may change during operation—most often deterioration—as a result of wear and tear processes. The effect of these processes is wear, i.e., “permanent, undesirable changes in the state of the rail element that is a part of the switch, occurring during operation in a continuous (railroad vehicle movement at different speeds), stepwise, or cumulative manner, as a result of which the period of fulfilling the given utility function by the element is permanently exhausted”.

Changes in the components of a facility, occurring during operation, can affect their surface layer of the spire and crosshead components and can usually be described by state characteristics whose values (*x*) are functionals:

(10) $$x_{i}=x_{i}[\;f_{i,a}\left(t\right)]$$

where: *x_i_*—*i*-th state feature, *f_i_*_,*a*_—mapping function for feature *i x* under a conditions, *t*—operation time.

**Figure 15:**
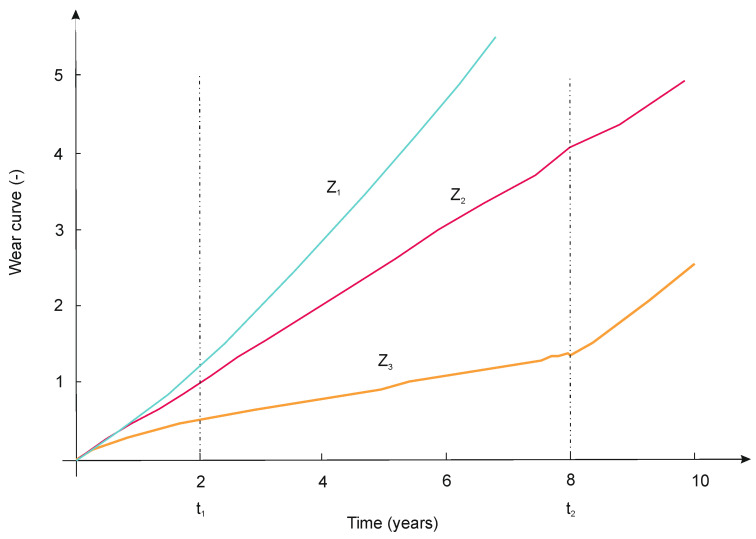
Wear curves for test data: z_1_—progressive, z_2_—proportional, z_3_—classic (Lorenz).

These features relate to the external structure of the examined railroad turnout components-the object of diagnostics. For many measurable features, their changes can be presented graphically, in the form of a wear curve. In the literature, among others in [51], three types of wear curves are distinguished (Figure 15):

− Classical (Lorentz curve);
− Proportional;
− Progressive.

From the point of view of railroad turnout operation, the best choice of wear curves is the classic (Lorentz curve). The classic (Lorenz) wear curve is characterized by the occurrence of distinct three periods in which the wear intensity changes. The first period, in which an initial high but decreasing wear intensity is observed, is characteristic of the process of component lapping. The course of the wear curve in this period (0 ≤ *t* ≤ *t*_1_) has a degressive character as a logarithmic curve. In turn, there follows a period of wear of constant intensity (linear), typical for normal operation (*t*_1_ < *t* ≤ *t*_2_). For the length of this period it is important that the proportionality factor *d* has a small value. In the third period (*t* > *t*_2_), the intensity of wear increases, which can be caused by increasing clearances or changes in the properties of the surface layer of elements. Since the wear curve in this area is progressive (exponential curve), there is a real danger of wear turning into damage-destruction.

The general relations describing the classical wear curve given in the literature [51] are insufficient for meeting the conditions of continuity and equality of its one-sided derivatives at the ends of the intervals determined by the operating times *t*_1_ and *t*_2_. These conditions will be satisfied if the course of the curve *z*_3_ is defined as follows:

(11) $$z_{3}=\{\begin{array}{c} a\cdot \ln\left(t+b\right)+c,when\;0\leq t\leq t_{1}\\ e+d\cdot \left(t-t_{1}\right),\;when\;\;t_{1}<t\leq t_{2}\\ h-f\cdot t_{x2}^{g}+f\cdot {\left(t-t_{2}+t_{x2}\right)}^{g},\;when\;t_{2}<t \end{array}$$

The coefficients appearing in Equation (11) are related as follows:

(12) $$\begin{array}{c} b=\mathrm{Exp}\left[\frac{-c}{a}\right]\\ d=\frac{a}{t_{1}+b}\\ t_{x2}={\left(\frac{d}{f\cdot g}\right)}^{\frac{1}{g-1}}\\ e=z_{3}\left(t_{1}\right)\\ h=z_{3}\left(t_{2}\right) \end{array}$$

The parameters of the coefficients *b*, *d*, *t_x_*_2_, *e* and *h* are the values read from the graph of the survival function for the different times of the railway turnout expansions. In the case under consideration, the classical Lorenz function is used. The values a and b are read off the graph of the curve used and are different for the time period analysed.

The actual wear curves prepared on the basis of measurements carried out during operation may slightly differ from the theoretical ones, which may be caused by the influence of variable load and exploitation conditions, and sometimes also by the damage incurred during disassembly and assembly of elements or by renovation methods (e.g., reduction of dimensions by machining). In each case, however, a real problem is to determine the limits of permissible changes of state characteristics that can be described by the wear curves and that are subject to assessment during diagnostic tests. This applies especially to the progressive and linear wear curve. In the case of the classical curve, the problem is basically solved because the moment *t*_2_ determines the end of the object’s life.

**Figure 16:**
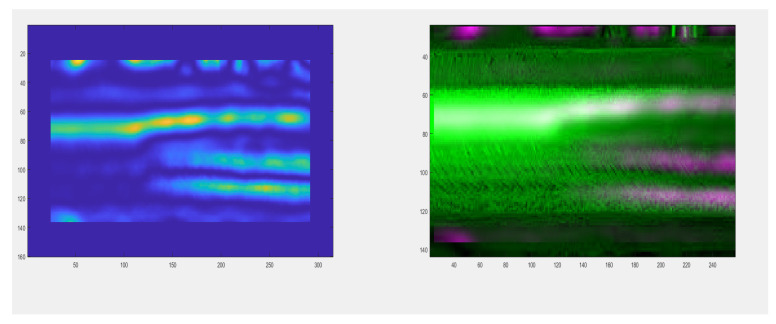
Wear on the rail of a railroad turnout determined using the algorithm developed by the authors.

**Figure 17:**
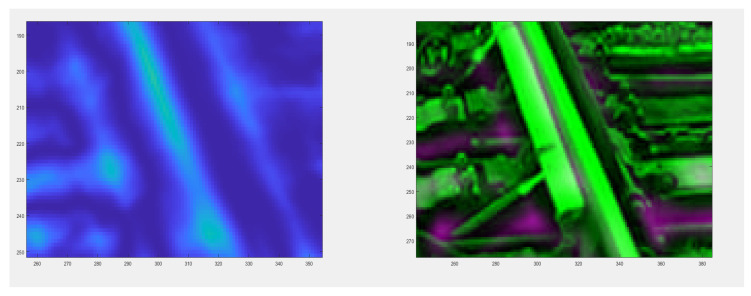
Example results of wear determination on a turnout needle.

**Figure 18:**
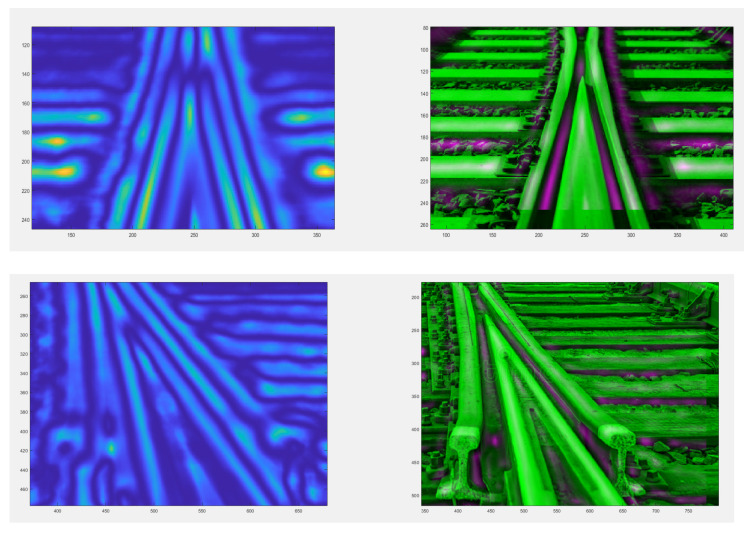
Example results of wear determination on a turnout cross member.

In the diagnostic process of the system developed for monitoring the technical condition of the railroad turnout, a significant problem is the classification of the detected wear on the railroad turnout in the analysed photographs. However, due to the difficulty of classifying all the elements subject to wear, a better solution is to apply the developed algorithm for wear identification on the basis of the photo analysis using modern image processing techniques. Considering the computational complexity of available software adapted to mathematical analysis and digital processing of photographs, it was decided that Matlab and Mathematica would be the most suitable numerical analysis environments. Four photographs were selected for test studies, where the spire was visualised in the first one and the cross beam in the next one, respectively. The computer simulations were divided into two stages. The first referred to the extraction of edges from the photograph based on the data obtained, in the later steps they will be compared with the reference photograph. As a result of the performed actions, the values of gaps and widths in the analysed segments of the railroad turnout were obtained, which are defined in Figure 16, Figure 17 and Figure 18.

**Figure 19:**
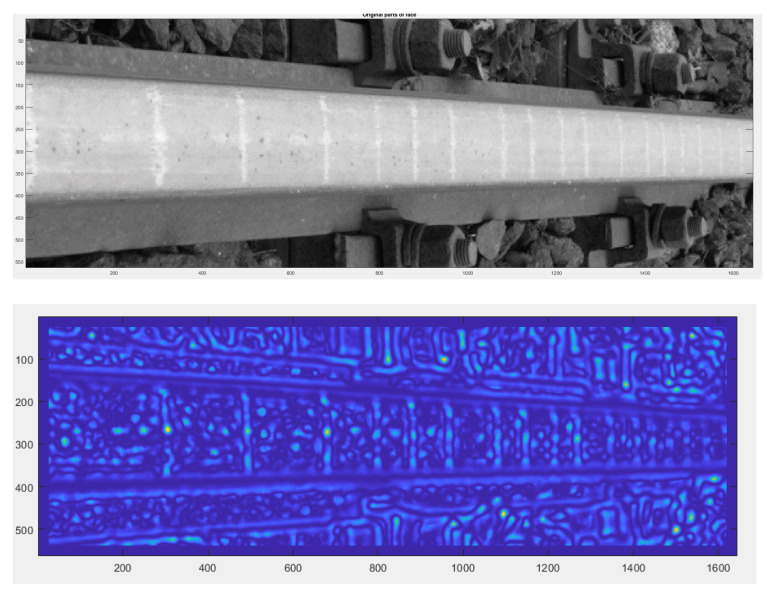
Example of the wear results of a main line turnout rail after 100,000 passes.

**Figure 20:**
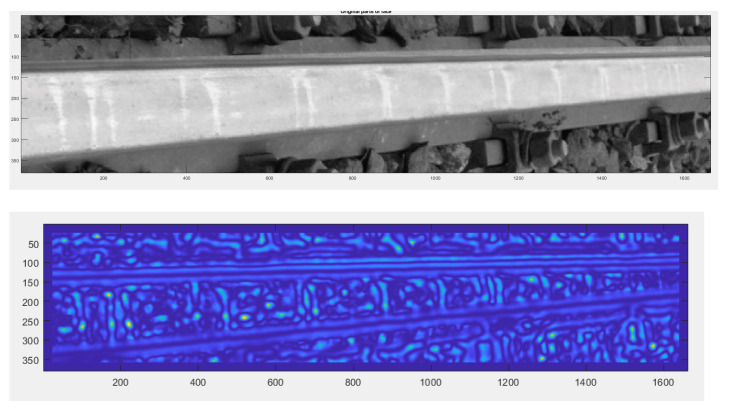
Example of the wear results of a main line turnout rail after 200,000 passes.

**Figure 21:**
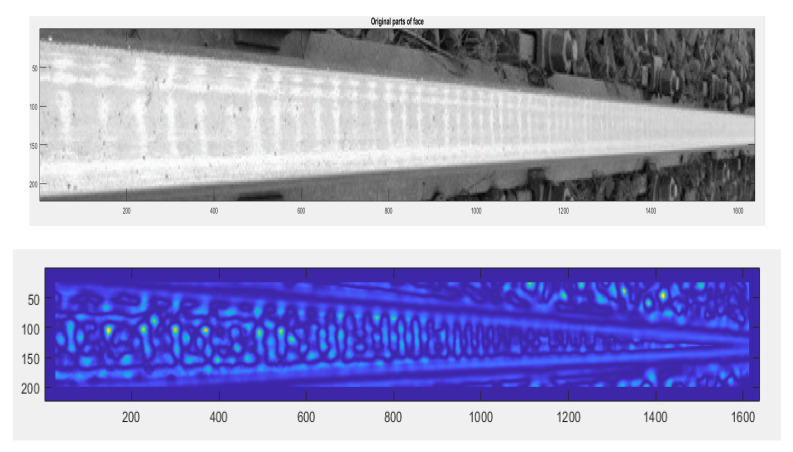
Example of the wear results of a main line turnout rail after 300,000 passes.

In a further test, the wear on the rail of a railway turnout was analysed for different values of passes of 100,000, 200,000 and 300,000. The results from these test runs are presented in Figure 19, Figure 20 and Figure 21.

The wear of the railway rails is shown in Figure 17, Figure 18 and Figure 19 and has been analysed using a script responsible for image analysis and processing. As a result, the areas on the surface where the material wear occurs have been marked. The greater the areas are, the more the colour changes from blue through yellow, orange and red. The results show cyclical wear of the rail resulting from the wheel–rail contact and the interaction between the wheel rims of rail vehicles and the rail head—its rolling surface and the inner edges of the rail track. It is a steel–steel contact during which there is friction along and/or across the rail head. Longitudinal friction usually occurs as a result of vehicle wheel slip during vehicle braking and acceleration. The result is vertical wear (reduction in height) of the entire cross section and damage visible on the running surface of the rail head. Transverse friction (pressure) usually occurs as a result of the phenomenon of rolling stock snaking (on straight sections) or as a result of the centrifugal force acting on the vehicle when negotiating a curve or turnout on a reversible track. Then the wheel flange comes into contact with the rail head resulting in the most characteristic phenomenon of lateral wear. The results of such measurements are recorded and processed electronically during or after the measurement and made available in the form of tabular (Table 5 and Table 6) lists of values of deviations of particular quantities or in graphical form, i.e., diagrams of the course of values of measured parameters on the axis of the travelled track (track, turnout).

**Figure 22:**
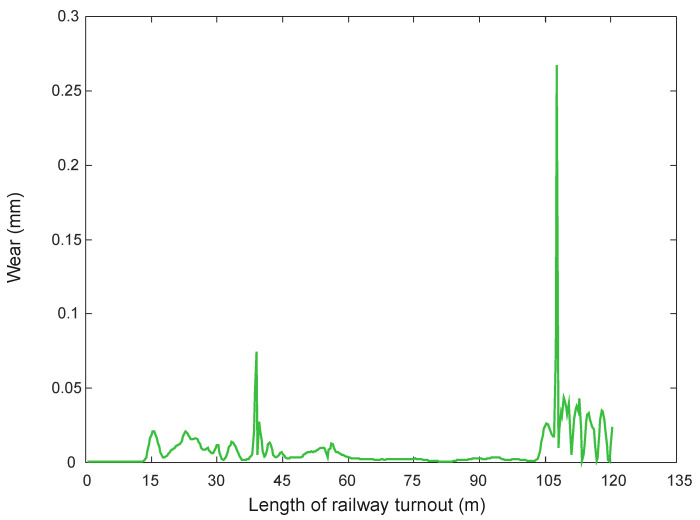
Railway switch wear determined after 100,000 rail vehicle passages.

**Figure 23:**
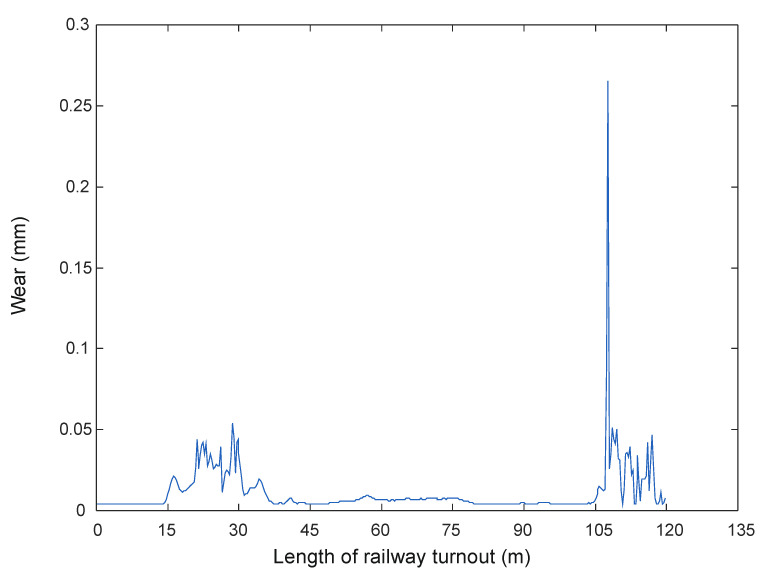
Railway switch wear determined after 200,000 rail vehicle passages.

**Figure 24:**
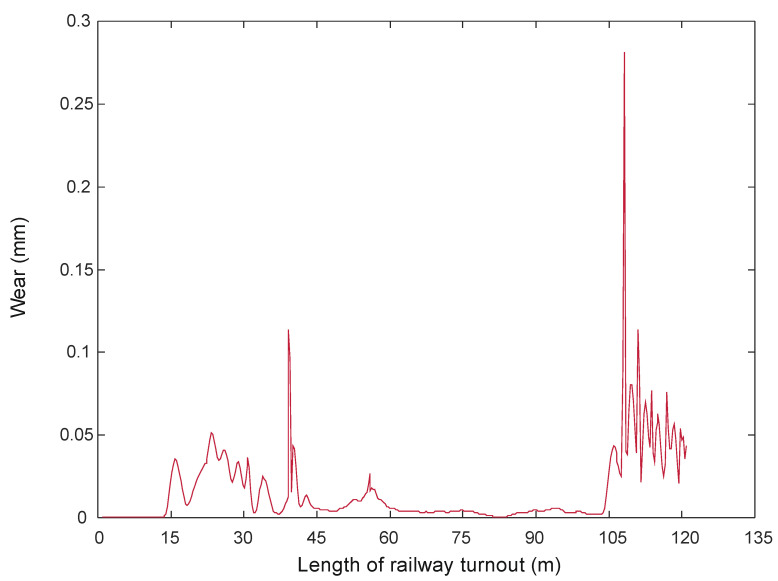
Railway switch wear determined after 300,000 rail vehicle passages.

The results in the form of wear patterns from the analysed images for the railway turnout are shown in Figure 22, Figure 23 and Figure 24.

The results obtained by the authors confirm that the rail wear in a railway turnout is influenced by its radius: the smaller the radius of the railway turnout, the greater the wear, and the course of this wear assumes a very sudden and irregular character. The speed of the moving rolling stock is also important: a higher speed causes an increased impact of the wheelsets on the outer rail in the form of wheel running up the edge of the rail of the turnout. The results show that a significant influence on the character and extent of rail wear of the railway turnout subassemblies has the direction of vehicle movement on the turning track crossing the cross track and the travel speed, which can be observed on the diagrams (Figure 22, Figure 23 and Figure 24). The greatest wear was observed at the beginning of the crossroads, reaching as much as 0.3 mm, while slightly smaller values were obtained at the spire, amounting to approximately 0.15 mm. On the presented results, a distinct irregularity of the path of the last car in tramway trains was observed, in the form of clear movements of the rolling stock car across the direction of travel. Such a phenomenon is the result of large increments in lateral acceleration, which results in large lateral forces transmitted to the rail and rail contact with the flange. These accelerations are greater the higher the speed of vehicle movement on a turnout curve. This phenomenon is aggravated by some drivers who, after the head of the train (their car and the first set of wheels) has passed through the curve, start to accelerate, as a result of which the last car of the train passes through the end of the curve at a higher speed than the first one. It is related to the specifics of the operating conditions occurring here and, as shown by the measurements carried out, to the irregularities in the geometric shape of the railway turnout and its structural elements.

## 7. Conclusions

The paper presents the problems of railroad turnout wear by simulating the passage of a passenger car through the turnout. The simulation results show that when a passenger car passes through the turnout at speeds above 250 km/h, the magnitude of turnout wear increases tenfold. These results indicate the need for online turnout diagnostics. 

The online turnout diagnostics system can also be applied to a larger number of diagnostic features and can concern both the whole objects and its individual elements. The measured values of individual features are the coordinates of the technical condition vector in the N-dimensional space. Apart from the difficulties related to the graphical representation of such a vector, it does not change anything in the way of interpreting the results of the measurements made during the diagnostic tests.

The possibility of using the developed method to determine the wear of railroad turnouts in the process of making operating decisions is independent of the type of wear curves, which can be used to describe important diagnostic features of the railroad turnout. An important difference between the features described by the proportional and classic wear curve is the way of establishing the limit values of these features. In the case of the classic curve, it is the increasing intensity of wear (for *t* > *t*_2_ as in Figure 13), whereas for the proportional curve, it is the conditions resulting from the cooperation with other elements (e.g., lateral contact of the wheel with the switch blade and the retaining cable).

## Figures and Tables

**Table 1:** Acceptable deviations of track gauge in a railway switch.

Track Gauge Deviations Permissible in the Switch Point [mm]			
v [km/h]	a|b|c|d	e	k|s
**160 < v < 200**	+4, −3	+4, −2	+4, −3
**140 < v < 160**	+5, −3	+5, −2	+5, −3
**120 < v < 140**	+6, −3		
**100 < v <120**	+6, −4		
**80 < v < 100**	+6, −4		
**60 < v < 80**	+7, −4		
**40 < v < 60**	+7, −4		
**v < 40**	+8, −4		

**Table 2:** Acceptable width deviations in the diverging line.

Track Gauge Deviations Permissible in the Diverging Line [mm]			
R[m]	b	c d	e k s
**190, 205, 230, 245, 265**	+8, −4	+14, −4	+8, −4
**300**	+8, −4	+10, −4	+8, −4
**500**	+7, −4	+9, −4	+7, −4
**760**	+7, −4	+7, −4	+7, −4
**1200**	+6, −4	+6, −4	+6, −4

**Table 3:** List of parameters measured in the turnout [71].

Measured Magnitude at the Turnout	Place of Measurement
Track width	In prespike contact a, in the switch blade b, in the firing pin socket c, in the middle of the turnout d, before the throat s in the cross member e,
The distance of the leading edge of the steering wheel from the proximal edge of the bow-f, the width of the groove:	In the firing pin seat g, at the handlebar h, in the cross member I, in the throat g and in the firing pin seat z

**Table 4:** Limiting values for rail usage criteria [28].

Turnout Class	Permissible Number of Rail Cracks per 1 km		Permissible Vertical Wear		Permissible Lateral Wear [mm]		Angle of Inclination of the Lateral Surface
	All	Original	UIC60 (60E1)	Other	UIC60 (60E1)	Other	Rail Heads
0	6	2	12		14		65°
1	7	4	14	8	18	12	
2	8	5	16	10	20	14	60°
3	9	6	16	14	20	17	55°
4 i 5	10	7	20	16	22	19	55°
lateral tracks	not specified		28	25	to the lower edge of the headstock		55°

**Table 5:** Wear values for a selected number of rail vehicle passes over the switch point.

Wear on the Switch Point of a Railway Turnout [mm]			
100,000 Crossings	200,000 Crossings	300,000 Crossings	400,000 Crossings
0.018894	0.003762	0.019488	0.004049
0.021184	0.006871	0.021491	0.0072
0.020783	0.010014	0.021008	0.010356
0.019032	0.012945	0.019184	0.013264
0.016417	0.015686	0.016363	0.015983
0.013358	0.017556	0.013486	0.017718
0.010308	0.016858	0.010642	0.017144
0.007181	0.014661	0.007694	0.015099
0.004781	0.011936	0.005435	0.012559
0.003674	0.009938	0.004638	0.010908
0.003644	0.008822	0.005068	0.010322
0.004122	0.008361	0.006377	0.01053
0.004825	0.008544	0.008134	0.011403
0.005715	0.009093	0.009962	0.012531
0.006809	0.009969	0.01175	0.013792
0.008031	0.010912	0.013192	0.01502
0.0089	0.01174	0.01452	0.016186
0.009638	0.012617	0.015636	0.017323
0.010196	0.013406	0.016807	0.018505
0.011146	0.014276	0.018012	0.019773
0.011947	0.022424	0.019186	0.018681
0.012379	0.039584	0.019953	0.003449
0.012988	0.025506	0.020235	0.000877
0.014816	0.021823	0.021371	0.014544
0.017411	0.02952	0.024472	0.024999

**Table 6:** Wear values for a selected number of rail vehicle frogs.

Wear on a Railway Turnout Crossing [mm]			
100,000 Crossings	200,000 Crossings	300,000 Crossings	400,000 Crossings
0.019021437	0.011179533	0.018715158	0.0115597
0.017684992	0.010821639	0.016486943	0.010957012
0.017259346	0.009673296	0.015502267	0.009711686
0.075947128	0.009099869	0.053248417	0.009000349
0.267199099	0.009619704	0.168566152	0.009842868
0.010054179	0.079236522	0.024390638	0.09651842
0.022788791	0.253174663	0.023171268	0.116597816
0.033589516	0.022028264	0.038722675	0.023451211
0.031664215	0.030807914	0.048212599	0.034961678
0.043442585	0.046030104	0.048400532	0.05198675
0.04211526	0.039790984	0.042300243	0.044810068
0.036907118	0.036804698	0.032132916	0.041637238
0.032383677	0.045607325	0.023893222	0.034509663
0.039810974	0.027712939	0.068443388	0.049289059
0.027858675	0.026957145	0.050509956	0.040222555
0.005392101	0.008609855	0.013282888	0.030161489
0.020745348	0.000609501	0.025899108	0.032789283
0.029234428	0.009533796	0.037988868	0.035321891
0.036362775	0.030835448	0.041910976	0.030259054
0.0376403	0.031145699	0.037576254	0.030210895
0.034243755	0.029213781	0.029209185	0.030362912
0.043150172	0.035401221	0.02573368	0.031871758
0.023274668	0.01872099	0.046133231	0.053801131
0.000924052	0.020947346	0.023791788	0.045672901
0.005102223	0.001089427	0.020386331	0.026517503

## Data Availability

The data presented in this study are available on request from the corresponding author.
